# A longitudinal investigation of the relationship between crowding and reading: A neurodegenerative approach

**DOI:** 10.1016/j.neuropsychologia.2016.02.022

**Published:** 2016-05

**Authors:** Keir Yong, Kishan Rajdev, Elizabeth Warrington, Jennifer Nicholas, Jason Warren, Sebastian Crutch

**Affiliations:** Dementia Research Centre, Department of Neurodegenerative Disease, UCL Institute of Neurology, University College London, UK

**Keywords:** Posterior cortical atrophy (PCA), Alzheimer's disease (AD), Dyslexia, Letter-by-letter reading, Visual crowding

## Abstract

We have previously documented two patients (FOL and CLA) with posterior cortical atrophy who achieved accurate and rapid reading despite deficits in ten measures of visual processing, with two notable exceptions: (1) a measure of visual acuity, (2) a measure of visual crowding. Subsequent longitudinal investigation of these patients was carried out, involving annual tests of early visual, visuoperceptual and visuospatial processing and assessment of reading ability. Follow-up assessments identified the evolution of a particular early visual processing deficit, excessive visual crowding; this deficit has been previously implicated in forms of dyslexia. Consistent with the link between crowding and reading dysfunction, follow-up assessments also revealed deterioration in both patients' reading ability. The current findings demonstrate a neurodegenerative approach towards understanding the relationship between visual crowding and the reading system, and suggest possible mechanisms for how excessive crowding may disrupt word recognition.

## Highlights

• Two patients demonstrate remarkably intact reading despite grave visual impairment.
• Longitudinal assessments of visual function and reading ability were carried out.
• Emergence of excessive crowding in central vision was observed in both patients.
• Emergence of crowding was accompanied by a decline in reading ability.
• Findings demonstrate a neurodegenerative approach towards acquired dyslexia.

## 1 Introduction

Visual crowding describes the inhibition of the identification of a target stimulus by the presence of flanking stimuli. This effect is primarily determined by the spacing between target and flanker stimuli, with reduced spacing leading to greater suppression of target identification; greater visual similarity between target and flankers also increases this suppression. In healthy individuals, crowding effects tend to be largely restricted to peripheral vision (with the critical spacing being proportional to the eccentricity; Bouma, 1970). However, in individuals with posterior cortical atrophy (PCA), a neurodegenerative condition characterised by progressive visual impairment, prominent effects have been observed in central vision (Crutch and Warrington, 2007, Crutch and Warrington, 2009, Yong et al., 2014a).

The occurrence of crowding when target stimuli and flankers are separately presented to different eyes indicates a cortical locus (Flom et al., 1963, Tripathy and Levi, 1994). Previous studies have placed this locus within the occipital cortex (Levi, 2008, Bi et al., 2009, Fang and He, 2008, Anderson et al., 2012), with functional localisation varying among V1 (Blake et al., 2006), V2 (Freeman and Simoncelli, 2011) and V4 (Liu et al., 2009). Functional imaging studies have identified an increase in crowding-modulated activation in the lateral occipital cortex (Chicherov et al., 2014) or from early to late visual areas (Anderson et al., 2012). Such findings have interpreted later visual areas being particularly involved in integrating or grouping features (Gori and Spillmann, 2010), and support crowding as a process that cannot be fully attributed to early visual areas such as V1. Correspondingly, there have been proposals of crowding as a multistage process, involving a lower-level feature detection stage, possibly in V1, and a higher-level integration of features downstream from V1 (Levi, 2008). In PCA, patterns of performance indicative of crowding when naming centrally-presented flanked stimuli have been associated with lower grey matter volume in the collateral sulcus (Yong et al., 2014a). While crowding tends to be considered a preattentive process, spatial attention may modulate crowding-related activation in early visual areas (Chen et al., 2014) and there have been suggestions that crowding itself arises from poor resolution of attention (Intriligator and Cavanagh, 2001).

Crowding is a promising candidate for a visual deficit that may fundamentally limit reading ability. Our uncrowded vision corresponds to the visual span (Pelli et al., 2007): the visual span is the extent to which we can read without moving our eyes, and has been proposed as a particularly significant factor in limiting our reading rate (Chung, 2004; Legge et al., 2001). Crowding might inhibit word recognition, parallel or serial letter processing through the interaction between the low-level features of words (Yu et al., 2012) such as letters (Martelli et al., 2005) or features of letters themselves (Fiset et al., 2008; Zhang et al., 2009). Excessive visual crowding has been proposed as a possible cause of developmental dyslexia, along with deficits in temporal and visuospatial attention (Franceschini et al., 2012) and diminished integrity of the magnocellular pathway (Gori et al., 2014); for a review of how these deficits relate to dyslexia including their neural and genetic bases, see Gori and Facoetti (2015). Developmental dyslexics have been found to exhibit particularly prominent crowding effects (Martelli et al., 2009, Pelli and Tillman, 2008) and increased interletter spacing has resulted in improved text reading performance of children with dyslexia (Zorzi et al., 2012).

Excessive crowding has previously been suggested to underlie a form of acquired dyslexia (Crutch and Warrington, 2009) and likely contributes to characteristic deficits in word recognition and text reading in PCA (Yong et al., 2014b, Yong et al., 2015). Two factors in the expression of crowding, spacing between target and flanker stimuli and visual similarity, may relate to specific reading deficits in peripheral dyslexia. One previously reported alexic PCA patient has been found to achieve optimal reading with words of moderate interletter spacing and lower letter confusability, a measure of the visual similarity of letters (Crutch and Warrington, 2009), while there have been suggestions of length effects in letter-by-letter (LBL) readers being artefacts of high letter confusability (Arguin et al., 2002, Fiset et al., 2005a, Fiset et al., 2005b).

We previously identified two patients (FOL and CLA) who maintained rapid and accurate reading despite impairments on ten measures of early visual, visuoperceptual and visuospatial processing (figure-ground discrimination, shape discrimination, hue discrimination, number location, dot counting, object decision, fragmented letters, canonical and non-canonical view perception, chequerboard experiment), establishing that such deficits were not sufficient to impair to reading dysfunction (Yong et al., 2013). These findings present a compelling challenge to general visual accounts of letter-by-letter (LBL) reading, which propose that reading is crucially undermined by even the most subtle visual deficits (Friedman and Alexander, 1984, Farah and Wallace, 1991, Price and Devlin, 2003; Behrmann et al., 1998). Exactly where such deficits might arise remains underspecified, with general visual accounts citing impaired peripheral, prelexical, early or general visual processing underlying LBL reading.

We proposed that FOL and CLA's efficient reading was maintained due to three factors: (i) their intact visual acuity; (ii) the relative preservation of the left fusiform gyrus, a region instrumental for orthographic processing (Roberts et al., 2012) and critically, (iii) an absence of crowding deficits when identifying centrally-presented flanked letter stimuli. Regarding condition (iii), neither patient made any errors on tests of centrally-presented flanked letter identification; furthermore, while both patients were slower than their respective control groups, neither showed the hallmark spacing effects, consistent across different flankers, that are characteristic of crowding.

The current study presents longitudinal data showing deteriorating reading speed and accuracy in both FOL and CLA. The main aim of this study was to investigate the evolving relationship between word recognition and crowding. It was hypothesised that any emergence of crowding effects with centrally-presented flanked stimuli would be associated with a deterioration in reading ability. Both patients began to exhibit flanked letter identification deficits consistent with excessive crowding at follow-up; the relationship between these deficits and reading is described below.

## 2 Methods

### 2.1 Participants

The study participants were the same two individuals with PCA as in Yong et al. (2013); FOL, a right-handed retired NHS administrator, and CLA, a right-handed retired classics teacher. At first assessment, FOL and CLA were 58 and 86 years old respectively. Nine control participants were administered the same tasks as FOL and CLA. The controls were split into two groups for each patient, matched for age, gender and years of education (FOL controls [N=4]: mean age 58.4 yrs [range 56–60], all female, mean education: 16 yrs; CLA controls [N=5]: mean 83.5 yrs [range 81–84], all female, mean education: 14.8 yrs).

#### 2.1.1 Imaging

**Fig. 1:**
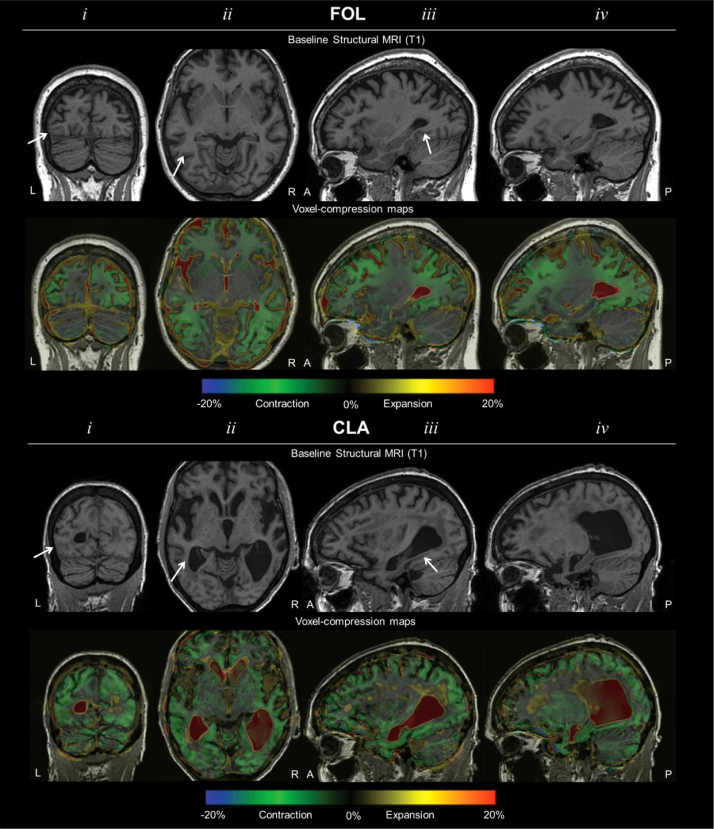
MRI sections and voxel-compression maps for FOL and CLA. (i) Coronal (ii) axial and (iii) left and (iv) right sagittal MRI sections for FOL and CLA at baseline and colour coded voxel-compression maps produced from subsequent scans (FOL: 25 months; CLA: 24 months), fluid-registered to baseline scans. A region within the boundaries of the VWFA as constituted by a functional imaging meta-analysis (Jobard et al., 2003) is indicated by the white arrows.

Fluid-based non-rigid image registration (Freeborough and Fox, 1998) was used to identify local volumetric changes in grey matter, white matter and cerebrospinal fluid between paired images from different time points (Baseline scans from FOL/CLA and follow-up scans at 25 and 24 months respectively). A viscous fluid model was used to calculate the warping or deformation needed to achieve correspondence of both images at the voxel level (Scahill et al., 2002). The Jacobian determinants of the deformation fields represent the location and extent of warping, and can be displayed as voxel-compression maps which show longitudinal expansion and contraction of local brain regions. The Medical Information Display and Analysis System (MIDAS) was used to overlay voxel-compression maps on rigidly aligned MRI scans for visualisation. Non-linear registrations of follow-up scans to baseline scans were performed for both patients; the resultant voxel-compression maps (Freeborough and Fox, 1998) are shown in Fig. 1. The white arrow indicates the mean activation peak of the visual word form area (x=−44, y=−58, z=−15) constituted from 17 functional imaging studies (Jobard et al., 2003).

FOL: Maps indicate relative sparing of left posterior fusiform (iii) and more extensive involvement of the right than the left occipital lobe.

CLA: While maps indicate diffuse atrophy, with extensive involvement of the occipital lobe, they also indicate the relative preservation of the left relative to the right inferior temporal lobe (iii).

#### 2.1.2 Background neuropsychology

FOL and CLA completed a battery of tests including a background neuropsychological assessment of memory, language, spelling and arithmetic and an assessment of early visual, visuoperceptual and visuospatial processing (see 2.2.1, 3.1: Visual assessment). Scores on each test are shown in Table 1. FOL was assessed 13 and 25 months after her initial visit, while CLA was assessed 17 months after her initial visit. CLA was due for a follow-up assessment at 31 months after her initial visit but was no longer suitable for testing due to a sharp deterioration in her condition, she did, however, complete the reading and crowding assessments at 27 months after her initial visit (see Section 2.2: Experimental procedures).

Across visits, FOL consistently demonstrated good performance on the concrete synonyms and spelling tasks. CLA also performed well on concrete synonym and spelling tasks for visits where background neuropsychological tests were administered (see Table 1).

### 2.2 Experimental procedures

Subsequent to the initial baseline assessment reported in Yong et al. (2013), the patients each completed two follow-up assessments (first follow-up [FU1] and second follow-up [FU2]), yielding a total of three assessments each. FOL was assessed 16 and 25 months after her initial visit, while CLA was assessed 18 and 27 months after her initial visit. Control participants only completed the baseline assessment.

#### 2.2.1 Visual assessment

Tests of early visual, visuoperceptual and visuospatial processing that were administered at the baseline assessment were repeated at follow-up assessments (see Table 1). Tests were administered at a distance of 30 cm.

##### 2.2.1.1 Early visual processing

(i) Visual acuity test from the Cortical Visual Screening Test (CORVIST; James et al., 2001): task required discrimination of squares, circles and triangles at decreasing stimulus sizes corresponding to Snellen form acuity levels.

(ii) Shape detection test from the Visual Object and Shape Perception battery (VOSP; Warrington and James, 1991): Figure-ground discrimination task involving random black pattern stimuli (N=20), half with a degraded ‘X’ superimposed. Patients were requested to state whether an “X” was present.

(iii) Shape discrimination: The stimuli (N=60) for this boundary detection task, adapted from Efron (1969), were a square (50×50 mm) or an oblong matched for total flux. There were 3 levels of difficulty: oblong edge ratio 1:1.63 (Level I), 1:1.37 (Level II), and 1:1.20 (Level III). The task was to discriminate whether each shape presented was a square or an oblong.

(iv) Hue discrimination (from the CORVIST): The stimuli (N=4) comprised 9 colour patches, 8 of the same hue but varying luminance and one target colour patch of a different hue. The task was to identify which patch was of a different hue.

##### 2.2.1.2 Visuoperceptual processing

(i) Object Decision (from the VOSP): Stimuli (N=20) comprise 4 silhouette images, one of a real object (target) plus 3 non-object distractors.

(ii) Fragmented Letters (from the VOSP): Participants were asked to identify visually degraded letters (N=20).

(iii) Unusual and usual views (Warrington and James, 1988): Participants were asked to identify with photographs of real objects (N=20) pictured from an ‘unusual’, non-canonical perspective. Items not identified from the non-canonical perspective are subsequently re-presented photographed from a more ‘usual’, canonical perspective.

##### 2.2.1.3 Visuospatial processing

(i) Number location (from the VOSP): Stimuli (N=10) consist of two squares, the upper square filled with Arabic numerals in different positions, and the lower square with a single black dot. Participants were requested to identify the Arabic numeral whose spatial position corresponds to that of the target dot.

(ii) Dot counting (from the VOSP): Stimuli (N=10) are arrays of 5–9 black dots on white background. Participants were asked to identify the number of dots without pointing.

(iii) A Cancellation (Willison and Warrington, 1992): Participants were requested to mark as quickly as possible with a pencil the location of 19 targets (letter As) presented among distractors (letters B–E) in a grid on an A4 sheet.

#### 2.2.2 Crowding assessment

FOL and CLA were requested to name aloud the same upper-case letters (excluding I, J, O, Q, W and X) as administered at the baseline assessment at follow-up assessments under the following conditions:

a. Unflanked letter identification (N=20): target stimuli were alphabetic items presented in isolation. Letters were presented in random order.

b. Letter flankers (N=24; e.g. ZNH): Target letters were flanked on each side by a letter, forming a 3-letter non-word combination.

c. Shape flankers (N=24; e.g. ◁N△): Target letters were flanked on each side by a triangle presented at different orientations. Triangles were of equal height and line thickness to target letters.

d. Number flankers (N=24; e.g. 6N5): Target letters were flanked on each side by an Arabic numeral, chosen from a range between 2 and 9.

In each flanking condition, target letter identification was probed under two spatial conditions, condensed and spaced. The edge-to-edge distance between the target letter and flankers was 0.1° in the condensed condition and 1.0° in the spaced condition, with the height of all stimuli corresponding to a visual angle of 1.0° at a viewing distance of 50 cm. The same combination of flankers was used for each target letter under both spatial conditions. The stimuli were presented in blocks of 6 items with the same spacing between the target letter and flankers, with blocks being administered in an ABBA design. All flanked and unflanked stimuli were presented in the centre of the screen within a fixation box (unflanked: 3.2° in width, 2.9° in height; flanked: 6.4° in width, 2.9° in height), ).

#### 2.2.3 Reading assessment

As previously, FOL and CLA were administered the Brown and Ure (1969) (N=72) words, the Schonell reading list (N=100; Schonell and Goodacre, 1971) and the Coltheart et al. (1979) regular/irregular words (N=78) yielding a total of 250 words ranging from 3 to 14 letters in length. Letter confusability ratings were averaged from the confusability matrices of van der Heijden et al., (1984), Gilmore et al., (1979), Townsend et al., (1971), and Fisher et al., (1969), with lower case ratings averaged from the confusability matrices of Geyer, 1977, Boles and Clifford, 1989. Letter height corresponded to a visual angle of 1.2° from a viewing distance of 50 cm.

### 2.3 Data analysis

FOL and CLA's performance at follow-up assessments was compared with control group data collected at baseline. Comparisons between both patients and their matched control groups were conducted using a modified *t*-test developed by Crawford and Garthwaite (2002) to identify abnormality of test scores in single case studies. For crowding and reading assessments, latency data for erroneous responses and responses where participants had become overtly distracted from the task were removed from the analysis. Latency data greater than 2 standard deviations from the mean of each participant were removed.

#### 2.3.1 Analysis of crowding assessment

Differences in accuracy of letter naming were compared between spacing conditions using a McNemar test. A separate logistic regression model for FOL and CLA was used to examine overall flanked letter identification accuracy, including spacing, flanker category and assessment as covariates, with robust standard errors to account for repeated presentation of stimuli across visits. As latency analysis was restricted to correct responses, high error rates at follow-up assessments meant crowding latency data were not analysed.

#### 2.3.2 Analysis of reading assessment

While neither FOL nor CLA made enough errors at baseline to allow for meaningful analysis of accuracy data, overall reading accuracy and latency analyses were conducted using logistic regression and linear mixed models respectively. The linear mixed model included as covariates assessment (baseline, FU1, FU2), word length, mean letter confusability, word frequency, and case (upper or lower), with random effects to allow for repeated presentation of stimuli across visits. The logistic model included as covariates the fixed effect variables from the linear mixed model, with robust standard errors to account for repeated presentation of stimuli across visits. Since CLA's performance was at ceiling at baseline, a small constant was deducted from her score at the initial visit, enabling us to provide a conservative estimate of the odds ratio between visits. As latency data for the reading assessment showed substantial right skew, statistical inferences were made using bootstrapped confidence intervals (95%, bias-corrected, accelerated with 2000 replications).

## 3 Results

### 3.1 Visual assessment

Consistent with baseline performance, both FOL and CLA continued to demonstrate impairments on all visual tasks except visual acuity which remained selectively spared (Table 1). The only other visual tasks on which FOL and CLA performed in the normal range at baseline (FOL: Object decision; CLA: Dot counting) both elicited impaired performance in both patients by FU1.

### 3.2 Crowding assessment

The number and percentage correct responses and mean and SD latency data for letter naming performance in unflanked and flanked conditions by FOL, CLA and their relevant control samples at each assessment are shown in Table 2.

#### 3.2.1 Single letter naming

FOL/CLA: Neither FOL nor CLA made any error responses at baseline, FU1 or FU2.

#### 3.2.2 Flanked letter identification

**Fig. 2:**
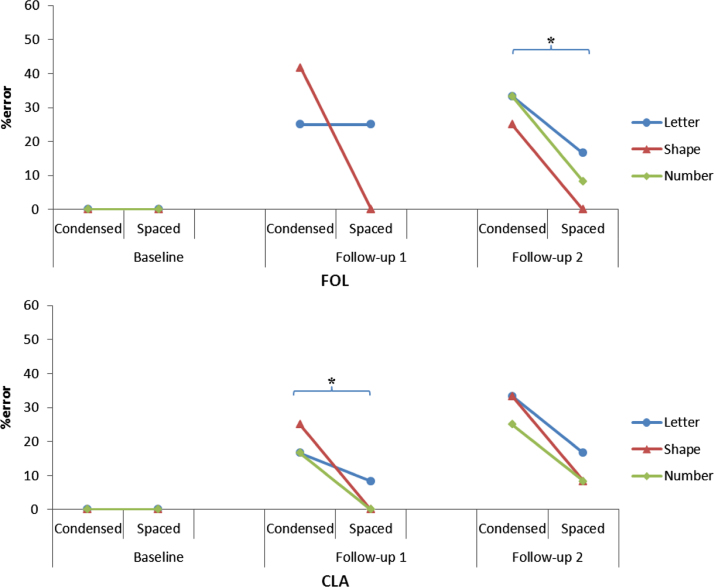
Flanked letter identification accuracy in condensed and spaced conditions across three longitudinal assessments (overall letter/shape/number flankers:*=p<0.05).

For mean percentage error rates across baseline and follow-up assessments, see Fig. 2.

FOL: FOL did not complete letter identification tasks in the number flanker condition at FU1. Overall analysis of FOL's accuracy data found that flanked letter identification was less accurate at subsequent assessments (z=−3.28, p=0.001). There was an overall effect of spacing, with letters being identified less accurately in the condensed condition (z=2.98, p=0.003). There was no significant interaction between spacing and assessment (p=0.31). There was no significant effect of flanker category on letter naming accuracy (letter vs shape flankers: p=0.11; letter vs number flankers: p=0.10; shape vs number flankers: p=0.79).

At FU1, while overall letter naming accuracy was poorer in the condensed relative to the spaced condition across letter and shape flanker conditions, this difference did not reach formal levels of significance using McNemar's test (66.7% vs 87.5%; p=0.18); in the shape flanker condition, there was a trend towards poorer letter naming accuracy in the condensed relative to spaced condition (58.3% vs 100.0%; p=0.063). At FU2, overall letter naming accuracy was poorer in the condensed relative to the spaced condition across all flanker conditions (69.4% vs 91.7%; p=0.033).

CLA: Similar to FOL, overall analysis of CLA's accuracy data found that flanked letter identification was less accurate at subsequent assessments (z=−3.53, p<0.001). There was also an overall effect of spacing, with letters being identified less accurately in the condensed condition (z=2.29, p=0.022). There was no significant interaction between spacing and assessment (p=0.94). There was no significant effect of flanker category on letter naming accuracy (letter vs shape flankers: p=0.43; letter vs number flankers: p=0.14; shape vs number flankers: p=0.53).

At FU1, overall letter naming accuracy was poorer in the condensed relative to the spaced condition across all flanker conditions using McNemar's test (80.6% vs 97.2%; p=0.031). At FU2, while overall letter naming accuracy was poorer in the condensed relative to the spaced condition, this difference did not reach formal levels of significance (72.2% vs 86.1%; p=0.27).

#### 3.2.3 Flanked letter identification error analysis

**Fig. 3:**
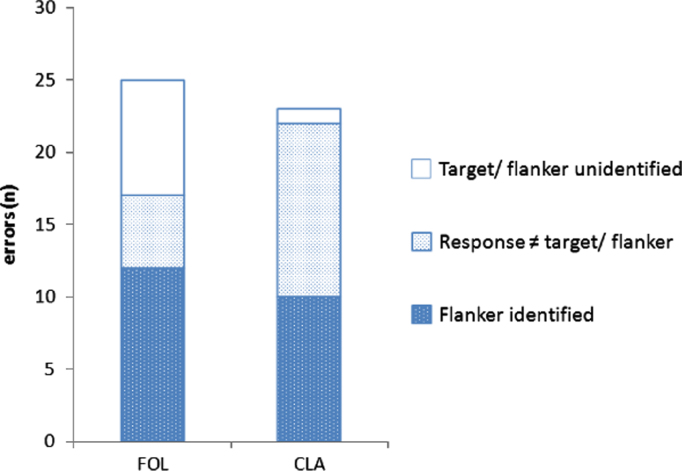
Flanked letter identification errors. Overall number of types of error made on flanked letter identification tasks.

The number of types of error responses made across both follow-up assessments is shown in Fig. 3. Error responses fell into three categories:

• Target remained unidentified, suggesting an inability to either detect or identify the target
• Response was a letter which was neither the target nor a flanker (e.g. YMT→V; 6F2→T)
• Flanker was identified rather than the target (e.g. ZNH→Z)

A greater proportion of FOL's errors were due to the target being unidentified, whereas a greater proportion of CLA's errors were due to responses which identified neither target nor flanker stimuli.

### 3.3 Reading assessment

**Fig. 4:**
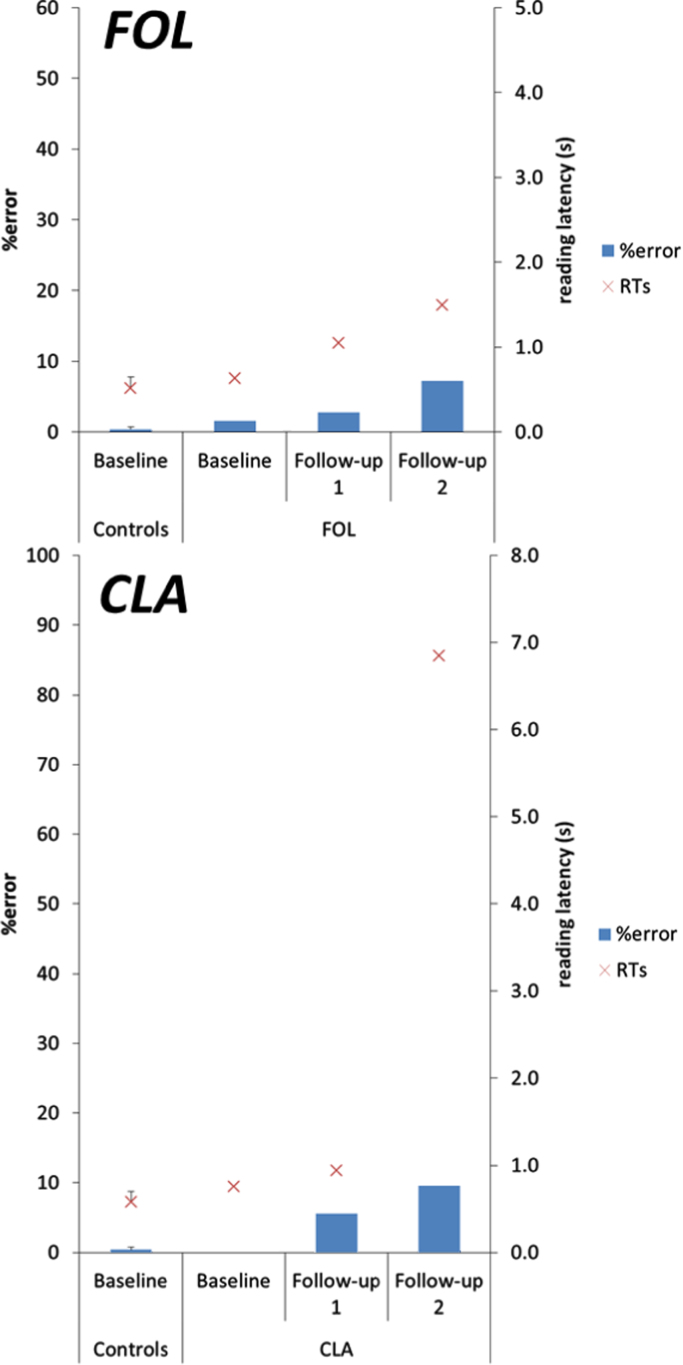
Overall reading accuracy and latency data across three longitudinal assessments. Error bars show standard deviation for control groups.

Mean percentage error rates and reading latencies for overall performance (summing across reading corpora) at baseline and follow-up assessments are shown in Fig. 4. Reading performance by FOL, CLA and their relevant control samples on the individual reading corpora is shown in Table 2.

#### 3.3.1 Overall reading accuracy

FOL: Overall analysis of FOL's accuracy data across the three assessments found accuracy decreased in subsequent assessments (z=−3.22, p=0.001). While there was a slight decline in FOL's overall accuracy between baseline (98.4%) and FU1 (97.2%), this did not reach formal levels of significance (p=0.18). However, there was a significant decline between baseline and FU2 (92.8%; z=−3.38, p=0.001) and between FU1 and FU2 (z=−2.26, p=0.024). Across the three assessments, longer words were read less accurately (z=−2.60, p=0.009). There were no significant effects of mean letter confusability (p=0.46), frequency (p=0.31) or case (p=0.18) on reading accuracy.

There was no significant difference in length effects on accuracy at follow-up assessments relative to baseline (FU1: p=0.42; FU2: p=0.55), however there was a trend towards longer words being read less accurately at FU2 relative to FU1 (z=1.72, p=0.085).

CLA: Overall analysis of CLA's accuracy data across the three assessments also found a decrease in accuracy in subsequent assessments (z=−5.38, p<0.001). There was a decline in CLA's overall accuracy between baseline (100%) and FU1 (94.4%; z=−2.79, p=0.005) and FU2 (90.4%; z=−3.39, p=0.001), with decline also evident between FU1 and FU2 (z=−1.97, p=0.049). Across the three assessments, longer words were read less accurately (z=−2.15, p=0.031) and there was a trend towards words in upper case being read less accurately (z=−1.84, p=0.066). There were no significant effects of mean letter confusability (p=0.49) or frequency (p=0.29) on reading accuracy. There were no significant differences in length effects on accuracy between follow-up assessments (all p>0.1).

#### 3.3.2 Reading error analysis

Errors were classified using criteria outlined in Crutch and Warrington (2009). Visual errors, defined as real-word errors in which≥50% of letters are maintained, made up 50% of FOL’s error responses at baseline, 57% of error responses at FU1 and 80% at FU2. CLA did not make any errors at baseline, whilst visual errors made up 60% of her error responses at FU1 and 65% of her error responses at FU2.

Of FOL's visual errors, 42.1% were deletion errors, 53.8% were substitution errors and 4.2% were addition errors. Of CLA’s visual errors, 23.1% were deletion errors, 44.3% were substitution errors and 32.6% were addition errors. Neither patient's visual errors showed an overall tendency toward being neglect errors; errors either showed no evidence of a spatial bias (FOL: N=10; CLA: N=11), were confined to the left (FOL: N=4; CLA: N=12) or right side of words (FOL: N=1; CLA: N=4; Ellis et al., 1987).

#### 3.3.3 Overall reading latency

**Fig. 5:**
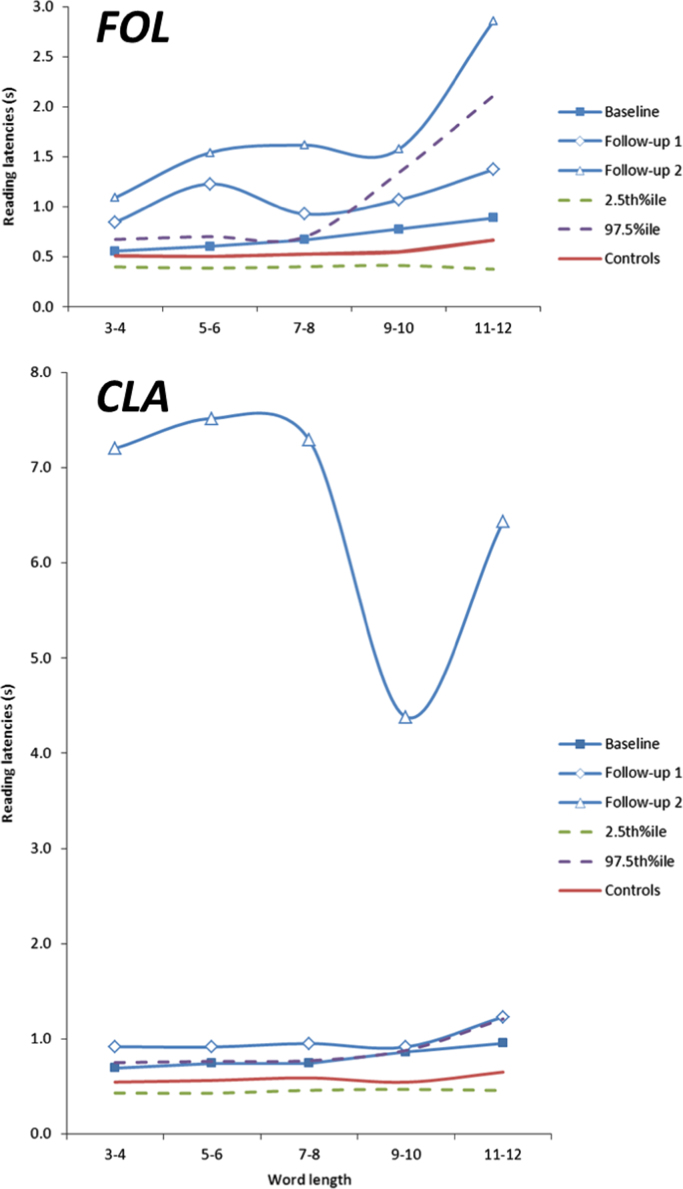
Latencies for words of different length. Overall reading latencies for words of different length are shown for FOL and CLA at different longitudinal assessments and baseline latencies are shown for their respective matched controls, with estimated upper and lower control confidence intervals.

Overall reading latencies for words of up to 12 letters read at baseline, FU1 and FU2 are shown in Fig. 5.

FOL: Analysis of FOL's latency data across the three assessments found that reading speed was slower in subsequent assessments. Reading speed was 0.63 s/word at baseline, 1.04 s/word at FU1 (95% CI [0.33,0.57]) and 1.49 s/word at FU2 (95% CI [0.67, 1.15]), with further decline in reading speed between FU1 and FU2 (95% CI [0.23,0.75]). Across the three assessments, increased word length led to slower reading speed (0.044 s word per additional letter; 95% CI [0.022,0.70]).

There was an interaction between word length and assessment. The effect per additional letter was 0.04 s/word at baseline and at FU1. At FU2, reading speed was 0.16 s slower per additional letter, a significantly greater effect than was observed at baseline (interaction 0.11 s/word 95% CI [0.004,0.29]). There were no significant effects of letter confusability (95% CI [−0.26, 3.45]), case (95% CI [−0.044,0.32]) or frequency (95% CI [−0.00023,0.00048]).

CLA: Analysis of CLA's latency data also found that reading speed was slower in subsequent assessments. Reading speed was 0.75 s/word at baseline, 0.94 s/word at FU1 (0.19 s/word slower than baseline 95% CI [0.12,0.24]) and 6.85 s/word at FU2 (5.96 s/word slower than FU1; 95% CI [5.09, 7.10)). There were no significant effects of word length (95% CI [−0.0063,0.11]), letter confusability (95% CI [−10.26, 4.37]), case (95% CI [−0.62,0.76]) or frequency (95% CI [−0.00095,0.0032]). There was no evidence of a significant interaction between length and visit.

## 4 Discussion

The current paper reports a two year follow-up evaluation of the relationship between visual processing and reading ability in two PCA patients, FOL and CLA. At the baseline assessment, despite showing comprehensive visual impairment, both patients demonstrated remarkably preserved reading ability, with CLA achieving 100% reading accuracy. The acuity of both patients was normal to near-normal, and neither patient showed evidence of excessive crowding when naming centrally-presented flanked stimuli. On follow-up assessments, while visual acuity remained relatively well-preserved, both patients showed a deterioration in their performance on centrally-presented flanked letter identification tasks, consistent with the evolution of prominent crowding effects. Concurrently, both patients’ reading speed declined relative to both their respective baseline performance and their age- and gender-matched control groups. At first follow-up, CLA was not only slower but also less accurate, making errors on high-frequency words (e.g., FREE→TREE, GLOVE→CLOVE). By second follow-up, reading accuracy for both patients was well below that of controls.

We hypothesised that a deterioration in reading ability would be accompanied by the emergence of excessive crowding, manifested through errors identifying centrally-presented letters flanked by letter, shape and number stimuli. The acuity of both patients was the same at baseline and first follow-up; assuming pupil size did not change, errors naming flanked letters at first follow-up would not arise as a consequence of overlap masking due to poor acuity. Consistent effects of spacing were observed in different flanker conditions, with elevated error rates resulting from targets with condensed flankers. This pattern of deficit is characteristic of visual crowding, and mirrors flanked letter identification deficits observed in other PCA patients (Crutch and Warrington, 2007, 2009; Mendez, 2001; Yong et al., 2014a). A previous eyetracking investigation of other PCA patients identifying the same centrally-presented flanked stimuli has suggested that this pattern of deficit arises even when patients are using central vision (Yong et al., 2014a, Fig. 4).

Having observed this co-occurrence of progressively diminished reading and heightened crowding, what is the nature of the reading impairment in FOL and CLA? Poor performance identifying flanked letter stimuli was not more pronounced with letter (i.e. same category) flankers as outlined under Shallice and Warrington's definition of attentional dyslexia (Shallice and Warrington, 1977; Humphreys and Mayall, 2001, Warrington et al., 1993). However, it is likely that deficits in spatial attention impact reading at and above the single word level, given the relationship between impaired visuospatial attention and reading acquisition (Franceschini et al., 2012) and how interventions which improve spatial cueing performance also result in improved text and pseudoword reading in dyslexic children (Franceschini et al., 2013; Gori et al., 2015). Visual errors comprised the majority of both patients' reading error responses, consistent with previous studies attributing acquired dyslexia to excessive crowding (Crutch and Warrington, 2009). Neither patient showed an effect of letter confusability on their reading ability; such an effect might be expected given how visual similarity modulates the expression of crowding. Both FOL and CLA showed poorer reading accuracy for longer words; this effect may be a result of increased numbers of letters exhibiting greater inhibitory flanker effects on parallel letter identification (Chanceaux and Grainger, 2013). In terms of reading speed, FOL showed not only poorer accuracy, but also slower reading speed for longer words; this length effect was more prominent at second follow-up. The “shrinking visual span hypothesis” (Legge et al., 1997) proposes length effects on reading latency might arise with words whose lengths exceed the size of the visual span, due to increased demands in the number of fixations. Excessive crowding would likely exacerbate such demands by reducing the visual span (see Pelli et al., 2007); however, such a reduction could also arise from a restriction in the effective field of vision which has been found to contribute to PCA patients' diminished ability to recognise large rather than small pictures, words and letters (Saffran and Coslett, 1996; Kartsounis et al., 1991; Coslett et al., 1995; Stark et al., 1997; Crutch et al., 2011, Yong et al., 2014b).

Variations in patterns of reading performance between the two patients may relate to differences in the quality of crowding effects between FOL and CLA. Assessment of reading ability identified how a greater proportion of FOL's error responses featured letter deletions; in contrast, a greater proportion of CLA's error responses featured letter additions. Assessment of crowding identified how a greater proportion of FOL’s errors arose from her being unable to provide a response to target stimuli, while CLA showed a greater tendency towards making error responses that named neither the target nor flanker. If flanked letter identification errors arise from competition between feature detectors, as proposed in lateral masking accounts (Townsend et al., 1971, Wolford and Chambers, 1984), crowding may limit letter detection as well as identification (Parkes et al., 2001; Pelli et al., 2004). By contrast, feature integration accounts suggest pooling of information over multiple features of flanker and target stimuli, inhibiting identification but not detection (Pelli et al., 2004; Greenwood et al., 2010). In the context of a two-stage model of crowding (Levi, 2008), FOL's lack of responses may reflect a low-level deficit in feature detection, whereas CLA's error responses may stem from a higher-level deficit of excessive integration between features of target and flanker stimuli. Whilst we cannot know the current distribution of pathology in our patients in sufficient detail, crowding as a failure of feature detection in FOL would predict greater pathological involvement of the striate cortex, while crowding as a consequence of excessive feature integration in CLA would predict disproportionate involvement of the extrastriate cortex.

In our previous investigation, we proposed that efficient reading in both patients was a consequence of preserved word form/parallel letter processing, maintained by the integrity of early aspects of the visual system (as suggested by strong performance on tests of visual acuity), the VWFA and interconnecting projections. In the current investigation, while both patients showed diffuse brain atrophy, we continue to argue for the relative preservation of the region corresponding to the VWFA at subsequent assessment. Given the dissociation between crowding and acuity (Song et al., 2014), even the sustained efficacy of both the early visual system and the VWFA might be undermined by enhanced crowding and any accompanying occipital atrophy. However, we cannot conclusively rule out the possibility that disease progression may have also compromised the structural integrity of the early visual system, projections to the VWFA or the VWFA itself based on imaging data from these two patients. Such interpretations would predict that constraints on basic visual input or disruption to orthographic processing would contribute to FOL and CLA's reading impairment. However, such interpretations might also predict effects of word length on reading latency consistent with previous reports of LBL readers (Mycroft et al., 2009); this notion receives little to no support from FOL and CLA's reading ability.

There were several methodological limitations to the current investigation. Each assessment used fixation boxes to support effective localisation of crowding stimuli, given both patients' deficits in visuospatial function. However, the fixation box may have exhibited crowding effects on flanked and unflanked stimuli, and the varying width of the fixation box between flanked and unflanked conditions may have created a potential confound. However, neither FOL nor CLA made errors naming unflanked letters. We advise that future investigations consider potential crowding effects exhibited by this presentation method; such effects may be mitigated using fixation cues positioned at greater eccentricities and/or of opposite polarity contrast to stimuli (Yong et al., 2015; Kooi et al., 1994). Reading corpora featured words in a font which varied in letter width and interletter spacing; we suggest future investigations of the relationship between crowding and dyslexia consider approaches to better control interletter spacing, for example, using fixed-width font. Future studies will also benefit from the development of nonletter stimuli to assess crowding in PCA. Crucially, such stimuli must exclude the need to make judgements based on identifying orientation or position, given PCA patients' frequent deficits in spatial cognition.

This longitudinal investigation follows the concurrent development of crowding deficits and declining reading ability in two patients with a neurodegenerative condition. These results further underline the relationship between crowding, a specific early visual processing deficit, and reading. The specificity of this deficit contrasts with the underspecified 'general visual impairment' which proponents of general visual accounts of reading regard as the causal component underlying acquired dyslexia. The current findings provide additional insight into different ways in which crowding may limit reading ability, and demonstrate a neurodegenerative approach towards understanding the relationship between one specific form of basic visual deficit and the reading system.

## Figures and Tables

**Table 1:** FOL and CLA's performance on background neuropsychological measures and tests of visual processing (not tested: NT). Shaded numbers indicate task performance is within normal limits (≥5th %ile).

*Test*	*Max score*	*FOL*			*C*LA		
*Baseline*	*FU1*	*FU2*	*Baseline*	*FU1*	*FU2*
**Background neuropsychology**							
MMSE^a^	30	24	23	15	27	13	*NT*
Short RMT words^b^	25	21	14	16	24	21	*NT*
Concrete synonyms^c^	25	20	21	20	20	20	*NT*
Spelling (oral)^d^	20	18	6	6	19	11	*NT*
Digit span (forwards): max	8	7	7	6	8	3	*NT*
Digit span (backwards): max	7	3	2	0	4	4	*NT*
**Visual assessment**							
*Early visual processing*							
Visual acuity (CORVIST^e^): snellen	6/9	6/9	6/9	6/12	6/18	6/18	*NT*
[Visual Angle equivalent at 30 cm viewing distance]	0.095°	0.095°	0.095°	0.134°	0.191°	0.191°	*–*
Figure-ground (VOSP^f^)	20	17	16	17	14	11	*NT*
Shape discrimination^g^	20	10	17	7	10	13	*NT*
*Visuo*spa*tial processing*							
Number location (VOSP)	10	5	0	*NT*	5	*NT*	*NT*
Dot counting (VOSP)	10	7	3	0	10	1	*NT*
A Cancellation^h^: completion time	90 s	60 s	90 s	90 s	50 s	90 s	*NT*
A cancellation: letters missed	19	1	6	16	0	6	*NT*
*Visuoperceptual processing*							
Object decision (VOSP)	20	15	14	13	7	*NT*	*NT*
Fragmented letters (VOSP)	20	8	5	1	0	*NT*	*NT*
Usual views^i^	20	18	20	*NT*	5	*NT*	*NT*
Unusual views	20	10	6	*NT*	0	*NT*	*NT*

a Mini-mental state examination (MMSE: Folstein et al., 1975).

b Warrington (1996).

c Warrington et al. (1998).

d Graded difficulty spelling test (GDST; Baxter and Warrington, 1994).

e Cortical visual screening test (CORVIST; James et al., 2001).

f Visual object and space perception battery (VOSP; Warrington and James, 1991).

g Efron (1969): Oblong edge ratio 1:1.20.

h Willison and Warrington (1992).

i Warrington and James (1988).

**Table 2:** (A) crowding assessment and (B) reading assessment accuracy and latency for FOL/CLA and their matched control groups; highlighted figures indicate where FOL/CLA's performance was poorer than their respective control groups (*=p<0.05;**=p<0.005, ^=*p* value unavailable using Crawford and Garthwaite (2002) modified *t*-test ceiling performance in control groups).

	A: Crowding assessment										
			*FOL*/*control raw scores*					*CLA*/*control raw scores*			
			*Control group*	*FOL*				*Control group*	*C*LA		
		*Max*	*Baseline*	*Baseline*	*FU1*	*FU2*		*Baseline*	*Baseline*	*FU1*	*FU2*
Single letter naming	Total	20	20 (100%)	20 (100%)	20 (100%)	20 (100%)	Single letter reading	20 (100%)	20 (100%)	20 (100%)	20 (100%)
RT (s)		0.48±0.06	0.59	*Not recorded*	1.08**	0.56±0.04	0.82**	0.87**	3.81**
Flanked letter identification	Total	72/48	72 (100%)	72 (100%)	**37/48 (77%)^**	**58 (81%)^**	Flanked letter identification	72 (100%)	72 (100%)	**64 (89%)^**	**57 (79%)^**
Condensed	Total	36/24	36 (100%)	36 (100%)	**16/24 (67%)^**	**25 (69%)^**	Condensed	36 (100%)	36 (100%)	**29 (81%)^**	**25 (69%)^**
Spaced	Total	36/24	36 (100%)	36 (100%)	**21/24 (88%)^**	**33 (92%)^**	Spaced	36 (100%)	36 (100%)	**35 (97%)^**	**32 (89%)^**

	B: Reading assessment										

1. Brown and Ure Words	Total	72	71.8±0.4 (99.7%)	72 (100%)	71 (99%)	71 (99%)	1. Brown and Ure Words	72 (100%)	72 (100%)	**70 (97%)^**	**69 (96%)^**
	RT (s)		0.51, SD±0.04	0.60	1.05**	1.66**		0.57,±0.06	0.64	0.91**	*Data missing*
2. Schonell	Total	100	99±1.0 (99%)	97 (97%)	97 (97%)	92 (92%)**	2. Schonell	99±1.2 (99%)	100 (100%)	94 (94%)*	88 (88%)**
	RT (s)		0.54±0.07	0.72	1.04**	1.58**		0.60,±0.06	0.78*	0.92**	6.52**
3. Coltheart Words	Total	78	78 (100%)	**77 (99%)^**	**75 (96%)^**	**69 (89%)^**	3. Coltheart Words	78 (100%)	78 (100%)	**72 (92%)^**	**69 (89%)^**
Regular	Total	39	39 (100%)	39 (100%)	**37 (95%)^**	**35 (90%)^**	Regular	39(100%)	39 (100%)	**36 (92%)^**	**35 (90%)^**
	RT (s)		0.48±0.04	0.56	0.97**	1.37**		0.53±0.05	0.91**	0.88**	6.62**
Irregular	Total	39	39 (100%)	**38 (97%)^**	**38 (97%)^**	**34 (87%)^**	Irregular	39 (100%)	39 (100%)	**36 (92%)^**	**34 (87%)^**
	RT (s)		0.51±0.05	0.59	1.15**	1.51**		0.55±0.05	1.1**	1.1**	7.87**
